# Responsible tourists in the time of Covid-19?

**DOI:** 10.1177/14687976231169559

**Published:** 2023-05-02

**Authors:** Bente Heimtun, Arvid Viken

**Affiliations:** UIT – The Arctic University of Norway, Norway; UIT – The Arctic University of Norway, Norway

**Keywords:** climate change, Covid-19, fatalism, neoliberalism, responsibility, tourism mobilities, tourist experience, travel habits

## Abstract

COVID-19 effectively stopped tourism mobilities for a time. Theoretically, this qualitative study draws on the notion of responsibility, as in responsibility to act and responsibility to Otherness. We explore how, during the pandemic, Norwegian tourists dealt with infection preventive measures, how they changed travel habits and how the pandemic transformed their thinking on tourism and climate change. The tourists were loyal citizens adhering to the authorities’ measures and refrained from international holidays, thereby taking responsibility for the governmentally enforced *dugnad* (collective efforts). This temporal change in travel habits, however, was not expected to become the new normal, as warmer, southern destinations were still desired. Culturally embedded neoliberal values of freedom of movement were, for most of these tourists, stronger than the threat of climate crisis. Fatalistically, we conclude that COVID-19 did not have the power to transform their mind-sets regarding responsible tourism futures and free them from neoliberal shackles.

## Introduction

In this article, we ask whether the COVID-19 pandemic has brought about new awareness of social and ethical responsibilities among Norwegian tourists. Before COVID-19, being a tourist entailed ‘the promise of mobility and freedom’ (Brouder et al., 2020), a freedom imbued with neoliberal ideologies founded on marketisation, privatisation, growth and consumer culture (Higgins-Desbiolles, 2020). Today, many Western democracies have adopted neoliberalism, a rationality governing all societal spheres ‘deeply ingrained into taken for granted everyday knowledge or common sense’ (Andreouli and Brice, 2021: 3). In Norway, before COVID-19, a decrease in air transport was not an issue (Guillen-Royo, 2022), and the freedom to travel was a cherished value. According to van Krieken (2020: 721),

The idea of mobility and extensive tourism—aiming to visit, no matter briefly, as many parts of the world as possible—has become central to many people’s sense of what it means to be a worthwhile human being, to be recognized by their social network.

COVID-19 was an effective showstopper for this lifestyle, which could also be labelled a ‘modern habitus’ of collective dispositions for widespread travel (cf. Linklater and Mennell, 2010). As neoliberal subjects, people’s major concern tends to be their own well-being, not care for other humans, climate and the planet (McEwan and Goodman, 2010).

The pandemic, tourism mobilities and climate change are closely linked. Because the virus travels with people, the pandemic has spurred a debate on whether and how the pandemic can be used to readdress climate change (Brouder et al., 2020; Gössling et al., 2021). Higgins-Desbiolles (2020) considered the COVID-19 crisis a moment of truth, revealing the many problematic sides of tourism and travel growth and tourism as a neo-liberal matter. Hall et al. (2020) argued that post–COVID-19 tourism will, in some destinations, develop in more sustainable directions, whereas in others tourism will seek to bounce back to the prepandemic situation. They expressed worry that many tourism stakeholders do not critically question the growth paradigm, thereby preventing a transformation from within. Others, focusing on tourists as individual customers, are even more pessimistic (Erickson, 2021), conveying what Solomon (2003) termed *fatalism*. This means that climate change is perceived as something we cannot master and even something the authorities tend to ignore (Bacevic and McGoey, 2021).

However, Stankov et al. (2020) stated that they trust the tourists’ mindfulness regarding their own practices, that tourists have a critical consciousness of the illusory promises of tourism that could entail value changes towards more basic experiences. They argued that ‘a mass mindful change’ on the part of tourists can lead to a more sensitive industry. Increased sensitivity also has been discussed as a desired mantra for contemporary tourism practices (Hurst et al., 2021; Viken et al., 2021), thereby addressing a higher level of responsibility towards the environment. Still, few studies have explored tourists’ contributions to responsible tourism (Caruana et al., 2014). In one such study, Eichelberger et al. (2021) argued that COVID-19 made tourists who identified as environmentally concerned wanting to make shorter trips, caring more for tourist communities, preferring sustainable transportation and avoiding mass tourism destinations in the future – thus, showing an increased will to act responsibly.

Against this backdrop, we asked in what ways Norwegian tourists have responded to state-imposed infection preventive measures, how COVID-19 transformed their travel habits, and in what ways the pandemic has increased their awareness of the impacts of tourism mobilities on climate change. Before we unpack the meanings of responsibility and responsible tourism, we introduce the Norwegian COVID-19 context.

## Norway and COVID-19

Acknowledging sociostructural factors, such as a country’s political and economic conditions, is important when seeking to understand the effects of COVID-19 (Jetten et al., 2021). Norway, a liberal democracy, values individual freedom. Well-known signs of neoliberalism are easy to identify (Mosedale, 2016): privatised national assets; commercialised parts of the public sector; creation of new markets; deregulation of the transport sector; and promotion of ideas related to innovation, entrepreneurship and market orientation. Part of this picture is also tourism, which is based on a well-developed transport system. Because of its sparse population and long distances between cities, domestic air transport is regularly part of many Norwegian people’s lives.

Norway is also an affluent, egalitarian and high-trust society, with a strong welfare state and a publicly financed national health care system (Bjørkdahl et al., 2021). This also includes a welfare mentality consisting of an alliance between state power and individual freedom, an alliance that is claimed to be stronger than in most other countries (Nilsen and Skarpenes, 2022). Still, several Norwegian governments, representing both social democratic as well as liberal conservative values, have implemented neoliberal policy measures during recent decades (cf. Evans and Sewell, 2013; Finstad, 2021). On March 12, 2020, however, the governmental strategies changed from liberalism to paternalism to manage the deadly health care crisis through laws, lockdowns, bans and guidelines (Askim and Bergström, 2022). Although ‘the approach was top-down and based on collaboration between political, administrative and professional central authorities’ (Christensen and Lægreid, 2020: 777), it was also marked by an atmosphere of cooperation and trust.

This atmosphere was enabled through a rhetoric of war and *dugnad* (collective efforts, also known as barn raising), appealing to Norwegian’s sense of solidarity (Bjørkdahl et al., 2021; Gjerde, 2021). The prime minister’s narratives on Facebook encouraged *dugnad* as ‘responsibilization and togetherness’ (Arora et al., 2022: 1). Montiel et al. (2021) labelled Norway’s rhetoric as universal collaboration: it promoted institutional responses and individual agency. This rhetoric ‘asserts an international identity committed to continue future action and responsibility. This bolsters egalitarian, collaborative relations, while also affirming accountability from govern bodies’ (Montiel et al., 2021: 762). This rhetoric of solidarity was also an expression of government control, followed up with disciplinary sanctions towards those not supporting the *dugnad*. (Neo)liberal Norwegian authorities exercised power by taking away inhabitants’ freedoms in regards of movement but not in protecting their health (Gjerde, 2021).

Through the rhetoric of *dugnad*, the prime minister appealed to a core moral value in the welfare mentality (Nilsen and Skarpenes, 2022): that special needs can be tackled by collective temporary efforts. Meta-narratives of infection-related protective measures in terms of ‘*dugnad*, solidarity and trustworthy leadership’ and, in part, ‘the dangerous-virus narrative’, also became part of people’s personal narratives on appropriate pandemic behaviour (Moss and Sandbakken, 2021: 894). People not taking this *dugnad* seriously were criticised. Yet, Norwegians coped differently with making sense of, and had varied emotional reactions and adaptations to, the policies and measures (Sandbakken and Moss, 2021). Not being allowed to travel, for instance, felt difficult but was understood. People acknowledged that ‘a change in lifestyle could be difficult’ (Sandbakken and Moss, 2021: 16).

## Responsibility and responsible tourism

Social responsiveness and responsibility are terms related to politics, morality and ethics and a topic in tourism analyses (Jamal and Higham, 2021). Here, we focus on responsibility, which can be defined in different ways. One way is backward-looking, labelled *ex post* or outcome responsibility, that refers to past actions (Miller, 2007; Pelenc et al., 2013). Having acted irresponsibly in the past can give reasons for penance in the future. During the pandemic, for instance, people were fined for breaking the infection-prevention rules. Also, the Norwegian prime minister was blamed for this (Bergh and Karlsen, 2022); when her husband hosted a restaurant birthday party for her, inviting family and friends, she was fined. More often, *ex post* responsibility is considered in analyses of failures and successes, whereby institutions accumulate knowledge and prepare for future decisions: ‘Institutions must acknowledge negative retrospective responsibility rather than attempting to hide it’ was Davies and Savulescu’s (2022: 3) reaction to how the British governments handled the pandemic. To prevent backward-looking accusers, and to gain trust, the Norwegian government acted responsibly by being open about the uncertainties of the measures applied during the outbreaks (Christensen and Lægreid, 2020).

In contrast, a forward-looking, *ex ante* responsibility enables ‘the agent to take others’ well-being (or obligations towards others, including non-humans) into account before acting’ (Pelenc et al., 2013: 88). Responsibility is then more than the act of doing. It is grounded in ethical thinking, guiding, for instance, tourists’ decision making (Fennell, 2008: 4). In what Fennell (2008) labelled the ethical stage of existence, the individual embodies and practises care for other people and society, thereby demonstrating a willingness to sacrifice that which is lovable and likable. This requires self-knowledge, questioning of one’s own ethical responsibilities, dealing with internal conflicts regarding choices and behaviour, and enacting values beneficial for the environment (Fennell, 2009).

White (1990) argued for a split between two interpretations of responsibility. The first relates to modernity and is ‘a modern-prudential obligation to acquire reliable knowledge and to act to achieve practical ends in some defensible manner’ (p. 80). This moral obligation derives from a rational need to survive, avoid harm and meet others’ expectations (Saarinen, 2021). Goodwin (2011: 16) defined responsible tourism in this way, claiming that it is to ‘create better places for people to live in and for people to visit’, adding that it also ‘recognizes the importance of cultural integrity, ethics, equity, solidarity, and mutual respect, placing quality of life at its core’. Here, responsibility connects sustainability to the tourist industry’s operational practices (Saarinen, 2021), which have been criticised for being an adaption to neoliberalism (Higgins-Desbiolles, 2020).

White’s (1990) second interpretation relates to postmodernism, whereby responsibility for the other is central and more in line with an *ex ante* responsibility. This moral strand is supported by scholars such as Lévinas et al. (1985), Taylor (1991) and Bauman (1991). Bauman maintained that the moral landscape or earlier meta-narratives that governed the individual, religion, ethics and secular rules, have lost much of their positions as compasses for good and bad behaviour in postmodernity. Still, Bauman argued, we are expected to follow norms, expectations and practices, acting responsibly. Goodhart (2017: 183) labelled this conventions. According to this way of thinking, people involved in tourism encounters should be sensitive towards each other (Butler, 2018; Viken et al., 2021).

Central to this notion of responsibility are moral-aesthetic concerns about otherness and our finitude, like the feminist moral philosophies of care ethics and the ethics of sustainability. These ethics emphasise how people act responsibly towards humans and nonhumans on power-imbued individual and collective levels (McEwan and Goodman, 2010). They equate people’s responsibility towards close as well as distant others and recognise people as relational and interconnected beings (Popke, 2006; Saarinen, 2021). Moreover, they reject the neoliberal notion of individualism and redefines the subject ‘as a collective, multi-layered, yet singular entity’ (Braidotti, 2006: 119). To Braidotti (2008), an ethics of sustainability is closely connected to becoming a person who endures and is faithful to sustainable changes and transformations together with others and the planet.

White (1990) further argued that modern and postmodern interpretations can or should be combined. Responsibility thus lies somewhere in between. In tourism, this is probably the most common approach. Smith (2009) ascribed responsibility to the individual’s ability to make critical judgements and to care for others, yet at the same time acknowledged that being ethical does not mean feeling guilty while enjoying tourism, thus allowing a focus on one’s own well-being and aims. Tourists’ enactments are connected to their identities, sometimes ideal identities, ‘holding ourselves under constant moral supervision . . . is not, as Freud recognized, good for our mental health’ (Smith, 2009: 273). Mkono (2020) also concluded that responsible tourists cannot escape feeling morally weak and frustrated when moral standards and action diverge. It is therefore not surprising that even environmentally concerned tourists experience attitude–behaviour gaps, justified through utterances like ‘It’s not that bad’; ‘It could be worse’; ‘It’s not my responsibility’; ‘I would like to, but’; ‘Vacations are an exception’; and ‘I am doing more good than bad’ (Juvan and Dolnicar, 2014: 86).

In addition to the modern and postmodern interpretations of responsibility, another understanding is related to neoliberalism. Here, responsibility is seen as a process of responsibilization ‘whereby individuals are ‘made responsible’ for their choices and actions. . .’ and forced ‘to “become responsible”, regardless of their convictions’ (Watts, 2022: 466). Brown (2006, 2015) argued that neoliberalism is more than an ideology underpinning an economic system; it is also ‘a mode of reason, a “political rationality” that today invades all spheres of life’ (Cornelissen, 2016: e12). As a rationality, neoliberalism has become the way we think and act, also related to responsibility.

Brown (2015) and Watts (2022) drew on the work of Foucault et al. (2008: 242), who redefined ‘*homo economicus* as entrepreneur of himself, being for himself his own capital, being for himself his own producer’. For Foucault, this ‘homo economicus is the ultimate market actor, one who seeks opportunities for self-investment at every turn’ (Watts, 2022: 467). In relation to Norway, Watts (2022) quoted Türken et al. (2016: 37–38), who argued that it constructs the individual as an autonomous subject who is encouraged to ‘take action’, ‘take personal responsibility’ and ‘work hard’ to achieve a ‘happy life’. These neoliberal ethics can also be found in contemporary tourism, through tourists’ self-gratification and pleasure seeking, being more like self-centred egoists or hedonists – seemingly without an explicit ethical value set (Fennell, 2009; Smith, 2009). In sum, we have identified three approaches to responsibility: modern, postmodern and neoliberal.

## Methodology and methods

We collected the qualitative data in North Cape, a major tourism destination and attraction in northern Norway, in September 2021, a few months after the government’s advice against domestic travel was removed. People still had to follow some infection-prevention measures. The data collection occurred before the omicron variant lockdown. We conducted the interviews in the cafeteria in the North Cape Hall, in the restaurant in a hotel in Honningsvåg, a nearby town where we also stayed, as well as in a local museum. Our rationale for choosing this location was that we expected to find Norwegian tourists visiting this hallmark attraction and destination (Hesla and Klo, 2019), during a period when only domestic travel was recommended. At all locations, we purposely recruited Norwegian informants.

We were inspired by a constructionist approach to interviewing as a situational, relational conversational practice cocreated by interviewer(s) and interviewee(s) (Brinkmann, 2013). Given this cocreation, our age and gender mattered (see Monforte and Úbeda-Colomer, 2021); we are one midlife woman and one man in late adulthood, thus having features similar to our interviewees (Table 1). Such shared circumstances enabled rapport. We applied somewhat different interviewer techniques that, combined, elicited engaging conversations in several interviews. The transcripts showed that interviewees asked questions that were interpretive, neutral and sometimes naïve (cf. Pezalla et al., 2012). We differed in that the female researcher’s questions were more often affirming and about including one’s own stories, whereas the male researcher was more often persistent and confrontational. The transcripts, however, also demonstrated that the female researcher, in the last couple of interviews, adapted her technique by becoming more confrontational. Our use of two interviewers thereby adds to the interviewer practices identified by Pezalla et al. (2012).

**Table 1.** Information list with pseudonyms, type of relationship, age group and number of interviewers.

Pseudonym	Type of relationship	Age group	No of interviewers
Man 1 Woman 1	Couple	67+	2
Man 2 Woman 2	Co-workers	50+ 67+	2
Man 3 Woman 3	Couple	50+	2
Man 4		50+	1
Man 5 Man 6	Co-workers	67+	2
Man 7 Woman 4	Couple	60+	2
Man 8 Woman 5	Co-workers	40+ 60+	1
Man 9 Woman 6	Couple	50+	2
Man 10 Woman 7	Couple	40+	2
Man 11 Woman 8	Couple	40+	2
Man 12 Woman 9	Couple	40+	2
Man 13 Woman 10	Friends	67+	1
Man 14 Man 15	Co-workers	40+	2
Man 16 Man 17	Co-workers	50+	1
Man 18 Man 19 Man 20 Man 21	Friends	50+	2

We conducted 13 dyadic interviews, thus consisting of two participants (Morgan et al., 2013); three interviews were done by one interviewer. We also did one group interview together with four participants and one individual interview (see Table 1 for a list). Our aim was to conduct all interviews together; however, in the search for participants in the sizable North Cape Hall, we sometimes split up. People interviewed together had preexisting relationships as couples, friends, or coworkers. These interviews enabled interactions and conversations between the interviewees, sometimes turning the interviewers into moderators. This mix of interviews was expected, given that most tourists have one or more travel companions. This required us to be flexible in the recruitment and during the interviews (cf. Morse, 1999). The average duration of the interviews was 30 minutes.

By doing predominantly dyadic interviews, we add to the limited amount of thinking about this approach in tourism research (Anantamongkolkul et al., 2019). Monforte and Úbeda-Colomer (2021) argued that coresearching and coreflections are positive for the research process yet noted that the use of two interviewers still has not received much attention in the literature. During the data collection, we had ample opportunities to discuss the interviews and our roles as recruiters and interviewers. Doing most of the interviews together also enabled fruitful analytical and interpretive discussions. Although the analysis was done by one of us, we both had in-depth knowledge about the conversations through conducting the interviews and reading the transcripts.

The conversational-styled, consented and audio recorded interviews were semi structured (cf. Brinkmann, 2013). When planning, we discussed ethical issues regarding anonymity and decided not to register names. We followed our university’s security guidelines related to the storage of recordings and transcripts. We also discussed questions to pose and decided on two sensitising concepts: (a) holidays taken before COVID-19 and (b) holidays during the pandemic, including a list of subtopics, depending on the interviewees’ experiences and reflections.

In the analysis, one of the researchers used the steps for thematic analysis recommended by Braun and Clarke (2006). All interviews were transcribed verbatim, followed by readings and discussions on the part of both of us to search for patterns related to responsibility, tourism and COVID-19. First, we coded the data set in Atlas.ti and, before moving the codes into a Word document, started the initial process of identifying themes through the software’s family function. At this stage, three themes were constructed: (a) infection protective measures (b) *Syden* (beach resorts in the Mediterranean) and travel abroad and (c) climate and mobility. In Word, all codes and extracts were systematised again into three partly new themes: (a) responsibility towards infection-prevention measures, (b) responsible changes in travel habits and (c) responsibility towards tourism mobilities and climate change. This refinement meant removing codes, moving codes around, recoding and grouping codes together into subthemes, focusing on supporting and contradicting coded extracts.

The study has some limitations. Because the data collection was done after the school holidays – in Norway, from mid-June until mid-August – there was a lack of young adult’s voices. Moreover, the Norwegian informants, shaped by the mentality of high trust and being familiar with the spirit of *dugnad*, meant that the findings may not be transferable to other types of societies. Our aim, however, was not to generalise our results but to unpack how our interviewees, at a specific time during the pandemic, dealt with infection prevention-measures in their holidays and how they reflected on this in relation to climate change.

## Findings: Caring for humans – ignoring the environment

In this section, we will explore the three identified themes.

### Theme 1: Responsibility towards infection preventive measures and guidelines

The interviewees were mainly loyal citizens taking seriously their responsibility for controlling the spread of the virus. By abiding with laws and regulations, they performed the simplest form of individual responsibility. Except for one interviewee, they had all abstained from travelling abroad after 12 March 2020. Although travel abroad was possible in the summers of 2020 and 2021, following governmental travel advice to engage in domestic holidays was considered a responsible choice.

Interviewer 1: You could have travelled abroad this summer?

Man 10: Yes, we could have.

Woman 7: We wanted it to be safer. Suddenly they lock down on short notice. If he [the husband] went into quarantine, it would be damaging for the shipping company.

Man 10: We have thought about, what if I ended up in quarantine, that would not be well received in the head office.

Moreover, uncertainties regarding frequently shifting advice from the authorities made them not want to leave Norway. In the first phase of the pandemic, when the government enforced the lockdown, travel bans and restrictions, which were communicated as the strictest restrictions since World War II, some informants felt petrified to the extent of not leaving their homes for fear of infection, fearing strangers in the local community and wanting all foreign tourists to go home.

Woman 2: When COVID-19 arrived, all tourists were perceived as very dangerous.

Man 2: They were seen as contagion bombs.

Woman 2: If you saw a foreign car, then it was put on Facebook – NB! NB! a German registered car outside Rema [grocery store].

With time, the informants accepted the rules and guidelines regarding face masks, hand sanitisers, social distancing, testing and quarantine. To keep in line with social distancing, some of them skipped visiting family members and friends in areas they passed through, and some avoided travelling to parts of Norway with high infection rates. Some informants struggled with how often to use hand sanitisers at the hotel breakfast buffet. Some always kept a mask in their pockets and clung to their private bottle of hand sanitiser: ‘it is a weapon that we have’ (Woman 8). One informant said ‘I hate the mask. I hope for God’s sake that it will end soon. But I understand why’ (Man 8). Two informants hiking in the northern parts of Sweden, when crossing the border to Norway in the wilderness, acknowledged the necessity of compliance and felt obliged to register this: ‘We wanted to do it right. We called [police] and stated our data. We promised to test when back home, and so we did. We waited three days in quarantine for the results’ (Woman 6). Just a few informants were fed up with the restrictions and the limitation of their freedom: ‘If I get infected, so be it. I think many think this way’ (Man 4).

Although most of the informants did not fear developing serious illness from COVID-19, they worried about infecting those at higher risk – family members, friends, co-workers and community members – thereby expressing a care for close and distant Others. Moreover, since the first lockdown, infection protective measures had become an embodied and disciplining habit imbuing their holidays and everyday practices, almost to the extent that it felt normal to consider other people as potential health hazards.

I notice how disciplined I have been: we disinfect ourselves at all points we come across, thinking about where you touch a door. You get a little disciplined, you stay on the slightly safe side. Walk around people a bit. Things you did not think of before. That thinking is very integrated here and now, all the time. (Man 15)

We were in Tromsø this weekend. When we went out to eat, if there were a lot of people, we went somewhere else. You just have to be careful. You can live quite normally. (Woman 8)

On the one hand, they trusted the authorities’ governance and recognised the difficulties of managing an unexpected crisis, accepted ‘trial and error’ in policies and measures and that authorities were open about not knowing exactly how to deal with infection control. On the other hand, the trials and errors, changes in argumentation and multiple lockdowns and re-openings also evoked negative reactions: ‘You become more sceptical, it seems that they are groping in the dark’ (Man 12). One short-term measure, which they obeyed, but did not make sense, was the banning of visiting second homes (*hytteforbudet)*, that in the first phase of the pandemic affected their personal freedom of movement:

Whether we got in the car and drove to the cabin and were inside the cabin or at home, it was the same. Because we met no one there anyway. So, it did not matter. They set a limit that we could not drive out of the municipality. We think that was a bit ridiculous. . . . Such rules make you frustrated and angry. We feel that it restricts our freedom. We, who live here in Norway and in other western countries, we are used to having enormous freedom. (Man 12)

Interviewees reflected on other people who paid less attention to COVID-19 measures: young people more interested in partying than social distancing, people sitting close in cafés and those going to *Syden* or leaving the country during the two pandemic summers.

Interviewer 2: Do you feel that you are taking social responsibility through the way you act? Is that an important side of it?

Man 9: I think it is very important to try to contribute. I have been embarrassed many times about being Norwegian when you read about people who do not know what to do when they cannot go to Sweden to buy cheap beer.

Woman 6: I get so nauseated from that.

The informants were split in their views on whether *Syden* tourists were egoists nor not. Some had followed this debate on social media. Here, people who left the country were described as selfish and were given the blame for the lockdowns. Some informants thought that leaving the country could be justified as people could have good reasons to go – for instance, to look after a property ‘Easy to say ugh, ugh, but I do not think that it is correct to do so’ (Man 12). Other informants characterised people as egoists. ‘We cannot leave it up to the individual to take responsibility, people go to *Syden* as soon as the opportunity is there’ (Man 19). Such thoughts were not only related to *Syden* holidays, but also destination substitutes in Norway. During the first summer of the pandemic, Henningsvær, a fishing village in the Lofoten Islands, was transformed into a hotspot for young Norwegian partygoers. People labelled it the Ibiza or Aya Napa of the north. Many young tourists disrespected social distancing and forced locals away.

Woman 6: We live in Lofoten and have observed the tourists. Last year was the first time that Norwegians came in a flock. We really got to experience how Norwegians are on holiday. Like in Spain, much alcohol. Henningsvær . . . people do not like it, but I call it the new Ibiza. This year they had to deploy police officers and guards.

Man 9: It took off completely.

Woman 6: Locals found drunk people in their basements. Drunk, people went into the houses and took a shower.

Thus, the interviewees saw the need for shared responsibility regarding the spread of the virus also on holiday. But first and foremost, they adhered to the government’s policies and advisories. They accepted the common responsibility. The rhetoric of the *dugnad* obviously worked –combatting the pandemic was something we had to do together.

### Theme 2: Responsible changes in travel habits because of COVID-19?

Before COVID-19, most of the interviewees holidayed abroad, to short- and long-haul destinations, once or several times per year. Many visited beach resorts in *Syden*. This habit was grounded in a desire and drive for spending time in a place with a guarantee of sunny and warm weather. Holidaying abroad was also described as a privilege and an identity marker for those born in the 1960s and 1970s. Thus, it was an embodied and culturally embedded convention. Not being able to continue this practice felt like a loss: ‘I am an abroad person who has been travelling between three and seven times a year: I miss it a lot’ (Woman 8).

Still, the pandemic was an eye opener for discovering and experiencing Norwegian destinations suitable for a variety of exciting nature activities. Only two informants did not change their habits; they had stayed at home or in their near-by second homes, as usual. A few of the sun lovers had realised that ‘it is possible to have nice experiences [in the county of Finnmark] even if the weather is not always good’ (Man 1), and that it was ‘fantastic to be here despite cold and rainy weather’ (Woman 9). Other informants had replaced *Syden* and international holidays with local hikes, local beaches and boat trips, and increased use of second homes: ‘This is also a holiday, you put away all that bother and stress’ (Man 15). One couple that had travelled abroad a lot used the pandemic to see most parts of Norway, including visits to the North Cape in the winter and late summer. They, however, longed for trips cancelled by the pandemic and expected that they ‘would travel less in Norway’ in the future (Woman 8).

Our data did not include systematic information about pre-pandemic domestic holidays. Moreover, it is too early to conclude whether the informants will change their habits post-pandemic. Although, *Syden* and many interviewees’ desire for sun, sand and sea are social constructs, the beach resort for them seemed to represented paradise and well-being. Thus, during the interviews, the enjoyment of *Syden* and other warm destinations lured, suggesting how such holiday habits have become engrained. One informant’s pragmatic rationale for wanting to travel abroad again was: ‘As we during the summer have received tourists from Italy, Germany and Switzerland, it must be possible for us to travel there’ (Woman 2). At the same time, the choice of destination would be carefully considered regarding infection rates, potential crowds of other tourists and measures and rules. Due to COVID-19 uncertainties, the interviewees were also cognisant of economical precautions in the booking process: ‘We will only book that which can be cancelled’ (Woman 2). In that regard, they were not crisis-resistant tourists, unaffected by the impact of COVID-19 upon holidays in the near future (Hajibaba et al., 2015).

Although several informants concluded that the pandemic and the measures would affect their lives and holidays for many years to come, just a few of them uttered hopes for the internalisation of new habits and that people would become more conscious about holiday destination choices (see also Sandbakken and Moss, 2021). Others were thinking that domestic holidays would be an add-on to *Syden*, and yet others would bounce back to pre-pandemic patterns: ‘I do not know if Covid can change conventions’ (Man 2). In fact, there seemed to be some fatigue among some informants, who wanted to reclaim their travel habits regardless of the pandemic ‘Eventually, you adopt a “don’t give a damn” attitude. I will travel anyway’ (Man 4), advocating the robustness of this lifestyle identity.

Thus, the pandemic had opened up new, more responsible tourism alternatives with the change towards more domestic holidays. Yet how the informants talked about this, indicated that it would not last – they missed holidays abroad. Travel habits and lifestyle patterns, thus, seemed hard to break. A *dugnad* is after all a temporary undertaking – which also underpinned the governmental rhetoric. In fact, the *dugnad* was an invitation to think of changes in holidaymaking as transient.

### Theme 3: Awakened responsibility for tourism mobilities and climate change?

Most of the informants just wanted to get back to the pre-pandemic normality and then continue travelling regardless of climate change, thinking that people would not change their behaviour much. Some talked about having become more aware of the consequences of their travel patterns and claimed a willingness to reduce international travel or travel more within Norway. The previous opportunity to travel everywhere had been a privilege that some feared had to change.

I’ve thought about it a lot, particularly in the beginning of the pandemic, when everything stopped. In India, they saw mountains that they had never seen before due to pollution.. . . I do not think we should travel as much as we have done. (Woman 6)

A few informants were also concerned with the sustainability of tourism mobilities in pre-pandemic times, acting responsibly during their holidays by practising biking, hiking and avoiding short-lasting holiday trips. They were hoping that more ‘people become aware of alternatives, not doing things on autopilot’ (Woman 5) and that tourism growth must come to an end to save the planet. ‘Everybody is saying – we must back to normality – but normality was not normal’ (Man 8), referring to the tremendous growth in travel pre-pandemic times.

In one interview, we had an in-depth discussion on this topic. One of the men had thought seriously about the impacts of his travel pattern and said it had been a luxury lifestyle that could not continue, even related to domestic holidays. He identified with pro-environmental norms and values, acknowledging the negative impacts of his travel patterns, and feeling responsibility to find an alternative behaviour. These thoughts were not shared by his travel companion:

Man 15: When I travel to Jotunheimen or Dovre [mountainous area in the middle of Norway], it’s three hours. I’m starting to think this luxury may soon be over. That thought strikes me. And I have a bad conscience when I go there.

Interviewer 1: So, you think about your footprint?

Man 15: Yes, a little, but what I think about more, is that I take it for granted to be able to travel 20–30 min just to have a weekend trip.

Interviewer 2: To have some fun?

Man 15: Yes. I have become so used to everything being within reach all the time. It is not certain it will be like this, 20 years ahead. One may have to holiday in nearby areas.

Interviewer 1: It sounds a little sad, if that happens?

Man 15: Maybe you need to take a train, maybe you need to walk a little bit further. It might be a little more like in the old days.

Man 14: I do not understand what you dream of.

The second man was curious about potential changes in tourism mobilities because of the pandemic, about whether it would increase or not. Yet, he concluded that he would continue his pre-pandemic travel pattern, despite acknowledging its impacts on climate change and hoping that his generation would take it more seriously. His rationale was being brought up in a travelling culture, thereby attributing his responsibility for climate change to others. His travel companion, however, did not think that environmental consciousness would change much, but still hoped that the pandemic could make a difference:

Man 15: For those of us who were born in the 70s, this is the normal.

Interviewer 2: The unlimited freedom, can it continue?

Man 14: We have not grown up with anything else.

Man 15: Yes.

Man 14: The world is so extremely much more open. We have grown up in a world where we can follow everything, we can get inspiration.. . . It would take a lot for me to change this when it reopens.

Interviewer 2: It’s so ingrained?

Man 14: I have been travelling during the holidays since childhood. Every summer has included some form of travel.

Interviewer 1: Abroad?

Man 14: Germany, France, car trips.

Interviewer 2: The journeys you make are unnatural.

Man 14: Is it unnatural for me who was born in 1977?

. . .

Interviewer 2: Is the pandemic nature running wild?

Man 14: The day we reopen, and it is safe to travel again, there will also be more reflections in my generation, on the climate and environmental aspects of travel. I think it will affect more than the fear of travelling in relation to the corona.

Man 15: I do not think so, I have no faith in environmental awareness, that it will make big changes. We’ve had it for a long time now.

Interviewer 2: We have changed.

Man 15: Not significantly when it comes to driving, travelling, consumption and littering. We try to make up for it by getting better at recycling and reuse and things like that, but consumption only increases and increases. Awareness needs something like the pandemic to land. So, you must relate it to something concrete. It is as if we are walking in a haze that makes us unable to relate to the reality that is there. The environmental challenges have been evident for so many decades. Something more is needed, and the pandemic has done just that.

During the pandemic, the informants accepted COVID-19 measures and acknowledged that the virus spread through tourism mobilities. In discussions on how the pandemic could be a lever for continued regulations of tourism mobilities in a post-pandemic time, as a means for reducing climate impacts, several measures were raised. Informants suggested prices as an effective regulating mechanism to balance out ‘ridiculously inexpensive’ air fares, yet also acknowledging the inequality of such measures – that it would affect districts in Norway and economies in countries dependent upon tourism. They also thought that many Norwegians were too well off to be discouraged by higher prices. Other ideas were related to reduction of airport capacities, taxation of unnecessary car travel mileage and societal value changes:

That domestic values are rewarded . . . Covid and climate are connected – that you contribute positively to the climate crisis by not travelling, as more young people do now. It is not only values, but also morality that comes into play. Do you travel, or not? (Woman 2)

However, they also thought people would react negatively if travel restrictions and regulations were imposed in post-pandemic times. One interviewee talked about how people, evolutionarily speaking, are egoists, not to be trusted as moral self-regulated individuals: ‘Increased travel needs to stop, it cannot continue, the big problem is that we are egoists . . . people want freedom to travel as they please’ (Man 10). In another interview, it was argued that labelling people as egoists could incite people to take more responsibility for saving the earth and atmosphere and thus travel less. However, it was also argued that political regulation of tourism and mobility after the pandemic would make Norway a totalitarian regime. What was needed was more sustainable transport alternatives:

I think that if you introduce regimes to restrict people’s freedom of movement, then you have something like a totalitarian regime. China is an example: if you have not behaved well, you will not be allowed to travel. Shame on you. It is better to work for sustainable travel alternatives, lower pollution from planes, cars and cruise ships. . . . Beginning with limiting where you can go, how long or how much you should fly or drive. No. That day, society is not good for anybody. It is totalitarianism. (Man 4)

The need for restriction on travel was acclaimed, primarily for the sake of infection control. Some informants admitted that this related to the climate crisis and the economic system behind it but hoped for a quick return of international travel. There were few signs of wanting a *dugnad* to reduce tourism mobilities. The freedom to travel is obviously strongly embedded. This indicated an antagonism between moral values, as aesthetics and ethics – something that has been addressed before – concerning travel and responsibility (cf. Fennell, 2008).

## Discussion

We have drawn on White’s (1990) understanding of responsibility as related to acting, a pragmatic use of the term, and responsibility for the Other, as a sort of moral imperative. Our findings showed that the Norwegian tourists took responsibility for controlling COVID-19, but as a pragmatic and situational concern. They were law- and norm-abiding individuals, thus, in line with White’s (1990) interpretation of responsibility of modernity. The tourists adhered to the government’s rhetoric of the *dugnad*, thereby contributing to combating the virus (cf. Moss and Sandbakken, 2021). By voluntarily participating in the *dugnad*, they did not harm or counteract the idea of freedom, which is central to neoliberal philosophy (Gjerde, 2021). They accepted a rhetoric that diminished the responsibility of the government, a pandemic strategy also known from other countries (Andreouli and Brice, 2021). Except for *hytteforbudet*, they did not consider much how this rhetoric was also imbued with government control and disciplinary sanctions (Gjerde, 2021).

The dangerous-virus rhetoric, intended to combat COVID-19, changed many of the interviewees’ holiday patterns from international towards domestic travel and avoidance of strangers, crowds and destinations with high infection rates (cf. Moss and Sandbakken, 2021). Avoiding visits to family members and fear of infecting others could be an expressions of *ex ante* responsibility and could be interpretated as a postmodern caring for the Other (White, 1990), in line with ethics of care (cf. McEwan and Goodman, 2010; Pelenc et al., 2013). However, it can also be interpreted as a ‘disruption of normality’, a term Andreouli and Brice (2021: 3) used to explain why people who would prefer to travel abroad, as responsible individuals, did not. After one and a half years of COVID-19, our participants had embodied infection-protection measures. They had internalised the conditions of the ‘new normal’ and adhered to governmental biopolitics (Gjerde, 2021).

Thus, for a while, COVID-19 changed most of our informants’ travel habits, and domestic destinations turned out to be positive experiences. However, they had not abolished their travel philosophy and priorities – many missed the warmth of *Syden*, a socially accepted destination for many Norwegians, wanting to temporarily escape a cold climate (cf. Guillen-Royo, 2022; Löfgren, 1999). Thus, the change was more a matter of law-abidingness than a consequence of ethical thinking and acting (cf. Fennell, 2008). There were few indications that those we met would give up the privilege of a Western neoliberal travel lifestyle (cf. Higgins-Desbiolles, 2020; van Krieken, 2020).

Our results contrasts with the findings of Eichelberger et al. (2021), indicating that COVID-19 had expanded people’s environmental awareness. However, both studies support the notion of tourism as a mental-free zone in which concerns for environmental impacts of tourism often are absent (see also Bernini et al., 2021). This echoes the old accusation against tourists as being ignorant and easily duped (Boorstin, 1962). It also supports the notion that even people with a strong drive to reduce the impacts of their tourism mobilities can find implementation hard (cf. Jacobson et al., 2020). The experiences of our tourists, thus far in the pandemic, seemed not to change their travel and tourism conceptualisation and become more mindful or sensitive, as Stankov et al. (2020) hoped.

Many of our informants thus did not commit to a shared responsibility for handling climate change. This supports Hall et al.’s (2020) prophecy that COVID-19 might not bring about long-lasting changes to the tourism system. It also reflects the profound embeddedness of the neoliberal ideology in a low-regulated, affluent society such as Norway. This ideology, envisioned by the government in the first phase of the pandemic – before the first lockdown – was obviously shared by many of the interviewees. Another interpretation could be that these people were ‘closing off sources of possible insights’ (White, 1990: 180) concerning the environmental impacts of tourism growth and how it has been one of the causes of the pandemic (McNeely, 2021). Although our participants behaved responsibly, most of them did not communicate a profound ethics of care or sustainability ethics for humans and nonhumans alike (cf. Pelenc et al., 2013); instead, they seemed to be behaving in accordance with the neoliberal version of the term, caring for themselves, their personal projects and own well-being, with extensive travel being normal. This assumption is consistent with the fact that 86% of Norwegians have a pragmatic, indifferent, or dismissive attitude towards climate and environmental issues (Livgard, 2021), not unexpected from people rooted in a neoliberal ideology and rationality.

The neoliberal thinking is massive. This is reflected in how our tourists thought about tourism during the pandemic. The neoliberal freedom was strongly ingrained, as it, for several decades, has been part of the governmental compass in Norway (Finstad, 2021; Gjerde, 2021). However, not only governments operate within neoliberalism; neoliberalism is also strongly rooted in international organisations and regimes (cf. Brown, 2015), such as the United Nations (UN), the International Monetary Fund (IMF), the European Union (EU) and the United Nations Tourism Organisation (UNWTO). Tremblay-Huet and Lapointe (2021) argue that a neoliberal rhetoric dominated UNWTO’s communication on COVID-19. In this rhetoric, responsibility was central, addressing individual as well as collective responsibilities. These responsibilities, however, can be hard to separate (Young and Nussbaum, 2011). Instead of banning tourism, UNWTO’s aim was safe travel, thereby upholding ‘the right to tourism’ (Tremblay-Huet and Lapointe, 2021). This organisation adjusted arguments and methods to an ideology where everything is ‘economicized’ and growth is a virtue. This suggests that COVID-19 pandemic has not been a game changer, not among the tourists, not among governments and not on the global scene.

## Conclusion

Our conclusion from this study, one and a half years into COVID-19, is a bit fatalistic. A sense of fatalism occurs when people lose control over an event and are incapable of acting (Solomon, 2003), which we argue happened in the pandemic and that, for a longer period, has been the case for the handling of climate change.

We have identified three types of fatalism. The first type is related to the Norwegian authorities’ lack of knowledge and their trials and failings – they were not prepared for and did not know how to manage a pandemic (Askim and Bergström, 2022). Other governments have also been blamed for this unpreparedness (cf. Bacevic and McGoey, 2021), despite the fact that pandemics and new diseases are not uncommon (Hall et al., 2020). The fact that the authorities were not better prepared could be explained by their governance, a sort of neoliberalism, termed fatalistic liberalism by Bacevic and McGoey (2021). The second type of fatalism is that our informants had accepted that COVID-19 was something they did not have any control over and with which they had to learn to live. This type is close to the origin of the term (Solomon, 2003). By September 2021, they had accepted governmental infection-protection measures and changed their travel habits significantly (cf. Moss and Sandbakken, 2021).

The third type of fatalism is that, despite knowledge about causes recently summed up by many scholars (cf. McNeely, 2021), it is difficult to see a trajectory that does not threaten the earth, its wildlife and humankind. Bacevic and McGoey (2021) labelled this epistemological fatalism. That science and technology have invaded and degraded ecosystems all over the world has been well documented and was addressed at the United Nations conference in Stockholm in 1972, repeated in the Rio Declaration, 1990, and later discussed in several United Nations–based climate assessment reports (Rusch et al., 2022). Thus, the relations among climate change, degradation of nature and eco systems and human health conditions and risks – including pandemics – are strongly evidenced (cf. Pandey et al., 2020). Despite this knowledge, the political ability to solve the global climate and environmental problems is continually demonstrated to be very low. We would argue that the power of neoliberalism – the ideology behind many of today’s climate challenges – is close to total. Globalised travel and tourism are parts of this, governed by a rationality and ideology that embraces freedom, consumption and pleasure. This was the reality of most of our informants: They did not critically reflect on the environmental aspects of their travel during the pandemic and were longing for free, unrestricted travel. Thus, most of them had adopted a neoliberal rationality (cf. Brown, 2015). In that sense, the political ignorance behind epistemological fatalism (Bacevic and McGoey, 2021) was shared by the governors and those they govern. The conclusion of this study is therefore somewhat depressing.

## Research Data

sj-docx-1-tou-10.1177_14687976231169559 – Supplemental material for Responsible tourists in the time of Covid-19? Click here for additional data file. Supplemental material, sj-docx-1-tou-10.1177_14687976231169559 for Responsible tourists in the time of Covid-19? by Bente Heimtun and Arvid Viken in Tourist Studies This article is distributed under the terms of the Creative Commons Attribution 4.0 License ( http://www.creativecommons.org/licenses/by/4.0/) which permits any use, reproduction and distribution of the work without further permission provided the original work is attributed as specified on the SAGE and Open Access pages ( https://us.sagepub.com/en-us/nam/open-access-at-sage).
