# Supplementary material for: Responsible tourists in the time of Covid-19?
Source: Tour Stud. 2023 May 2;23(2):87–107. doi: 10.1177/14687976231169559 (PMC10158805; doi:10.1177/14687976231169559)
Supplement: sj-docx-1-tou-10.1177_14687976231169559 – Supplemental material for Responsible tourists in the time of Covid-19? [file sj-docx-1-tou-10.1177_14687976231169559.docx]

# Research data statement

The data for this paper cannot be shared now. It will be used in further publications together with new data, collected in the four-year long project. The data will be achieved, with limited access, in Norwegian Centre for Research Data by 10.12.2025
